# Glances at the Hospitals

**Published:** 1903-10-10

**Authors:** 


### GLANCES AT THE HOSPITALS.

### THE CORK STREET FEVER HOSPITAL, DUBLIN.

The receipts were £9,900 and the expenditure was £11,631, showing that the account was considerably over-drawn. During the year arrangements have been entered into to enable the nurses to join the Royal National Pension Fund for Nurses, the hospital paying half the premium. This is a most excellent example and one which we should like to see widely followed. It is curious to note that while the number of enteric fever cases has greatly diminished of late years, the number of diphtheria cases has increased. In 1901 there were 195 cases fewer of enteric than in the previous year, and in 1902 there were 85 cases fewer than in 1901. The types of disease has, however, been severe, and shows a mortality of nearly 10 per cent. On the other hand, the diphtheria cases have risen from 89 in 1899 to 119 in 1900, to 123 in 1901, and to 167 in 1902. The hospital is in need of small wards for the treatment of mixed infection or for sporadic cases of typhus. At the beginning of the year the hospital contained 112 patients, and 1,456 were admitted, making a total of 1,464 under treatment during the year. Of these 1,317 were discharged, 147 died, and 104 remained in the wards. Taking all the cases under treatment, the mortality for the year was just a fraction over 10 per cent., but, deducting cases admitted in a hopeless condition, it would be reduced to 8·12 per cent. The tables are well compiled, and each one gives a synopsis of its contents put in a single line. Glancing at these tables we find that 123 cases of enteric were admitted, that 12 died, being a mortality of 9 75 per cent. There were 175 cases of pneumonia, and 22 of these died, being a percentage of

12·50. Of 317 cases of scarlatina 19 died, being a mortality of 5·98 per cent. Of 167 cases of diphtheria 19 died, being 11·37 per cent.

ROYAL VICTORIA HOSPITAL, BOURNEMOUTH.

The receipts amounted to £3,094, and the expenditure to £3,374, showing an adverse balance of £280; and there was a debit balance of £92 standing over from the previous year. The cost per head per week was £1 0s. 10¾d., and the average cost of each in-patient was nearly £6. The total number of in-patients was 512. Of these 317 were discharged cured, 82 relieved, 50 were incurable, 20 died, and 42 remained in the wards. The average duration ef each patient's stay in the hospital was 24 days. From the operation table we learn that ovariotomy was performed five times, all being successful; similar results followed the operation for the radical cure of hernia in 24 instances, and in 19 cases of appendectomy. There were 8 cases of removal of the thyroid gland and of these 2 died. A medical table is furnished, and there is also a short table showing the nature of the accidents admitted during the year. Nothing is said about the nature of the anæsthetics used during the operations.

WATFORD DISTRICT HOSPITAL.

The income was £810 and the expenditure was £883, showing a balance of £73 on the wrong side. The governors have bought an adjoining property for £1,000, and new wards are being built at a cost of £3,500. The additions will include committee-room and dental rooms. One of the new six-bedded wards will have fair cross-ventilation; but the other has not, and it is to be regretted that the faults in the old wards should be perpetuated in the new ones. The total number of patients treated was 350. There were 130 operations, 32 medical cases, and a total of 6 deaths. A very carefully-prepared table shows the sex, age, and previous occupation of the patients, also the nature of disease or injury and the results. Such a table is eminently useful in finding the issue of any particular case, but it is not so good for finding the collective results at a glance as properly prepared medical, surgical, and operation tables would be. The anæsthetics used are not mentioned.

THE STOCKPORT INFIRMARY.

The ordinary receipts amounted to £4,348, and the ordinary expenditure to £5,192, showing a deficit of £800 on the year's working; and the previous year there was a deficit of £1,000. Legacies to the amount of £861 were applied to reduce the debt, and by this means the adverse balance was brought down to £625. These figures would show a deficit on the two years of £939 and not £625; but it is probable that legacies were placed to the ordinary income in the year 1901 as well as in 1902. The revenue for 1902 was higher than in the previous year by £200, and it is satisfactory to notice that the workpeople's own subscriptions were £85 more than in the previous year. During the year 766 in-patients were admitted, and there was a total of 832 under treatment. Of these 610 were cured, 120 relieved, 51 died and 51 remained in the wards. The medical and surgical tables might almost as well have been omitted, as they do not give results. The operation table shows the same deficiency, which is a pity, as 253 operations were performed. There is no anæsthetic table.

